# Boiling histotripsy method to mechanically fractionate tissue volumes in e*x vivo b*ovine liver using a clinical MR-guided HIFU system

**DOI:** 10.1186/2050-5736-3-S1-O88

**Published:** 2015-06-30

**Authors:** Vera Khokhlova, Ari Partanen, Adam Maxwell, Tatiana Khokhlova, Wayne Kreider, Michael Bailey, Navid Farr, Yak-Nam Wang, George Schade, Oleg Sapozhnikov

**Affiliations:** 1 University of Washington, Seattle, Washington, United States; 2 Philips Healthcare, Bethesda, Maryland, United States; 3 Moscow State University, Moscow, Russian Federation

## Background/introduction

Most current HIFU approaches to treat liver tumors rely on thermal tissue ablation. Challenges still remain that prevent widespread clinical application of this technology including long treatment times, side effects such as skin burns, attenuation and aberration by ribs, heat diffusion and perfusion. Recently, a new method named boiling histotripsy (BH) was developed at the UW/MSU to address these challenges. BH applies a sequence of millisecond-long pulses with high-amplitude shocks that rapidly heat tissue and initiate boiling within each pulse. Interaction of shocks with the resulting vapor cavity leads to tissue fractionation into subcellular debris. Multiple BH lesions can be generated to sonicate clinically relevant tissue volumes. The goal of this study was to evaluate the feasibility of a clinical HIFU system to treat tissue with BH and to develop exposure protocols for such treatments with real-time and post-treatment MR imaging.

## Methods

Experiments were conducted with a 256-element 1.2 MHz HIFU phased array (Sonalleve, Philips Healthcare, Vantaa, Finland). Sonications were performed in *ex vivo* bovine liver at a tissue depth of 2 cm with 10 ms-long pulses and pulse repetition frequencies (PRFs) of 1 – 10 Hz. Pulses were delivered at 250 W acoustic power providing a 65 MPa *in situ* shock amplitude. Using electronic steering transverse to the beam axis, volumetric lesions were generated by treating circles of 2, 4, 6, and 8 mm radii, with 2 mm separation between focal points along each circle and 5 mm separation between the circles along the beam axis.

Two treatment protocols were tested: a sequential treatment with a set number of pulses delivered at each target location before proceeding to the next location, and a non-sequential treatment with consecutive HIFU pulses sent to different target locations to diminish heat accumulation and thermal effects. For all treatments, each point received 30 pulses. MR imaging was used to monitor treatments in real-time as well as to characterize lesions post-HIFU. Finally, lesions were also analyzed grossly and histologically.

## Results and conclusions

It was shown that the clinical HIFU system was capable of producing mechanically fractionated volumetric lesions in tissue using boiling histotripsy combined with electronic steering of the HIFU beam. The lesions were visible by MR both in real time and post-treatment. Successful sonications performed at 2 cm depth in tissue required less than 25% of the maximum system power, thus permitting implementation of this approach under clinically relevant conditions with greater attenuation. Homogenized lesions of 3 – 5 cm3 were produced with a 1 Hz PRF while increasing thermal effects were observed for sonications at 3 – 10 Hz PRF. With a 2 mm lesion separation, adjacent lesions merged to produce a single volume of fractionated tissue. It was observed that larger vessels could be spared while surrounding liver tissue was effectively fractionated.

**Figure 1:**
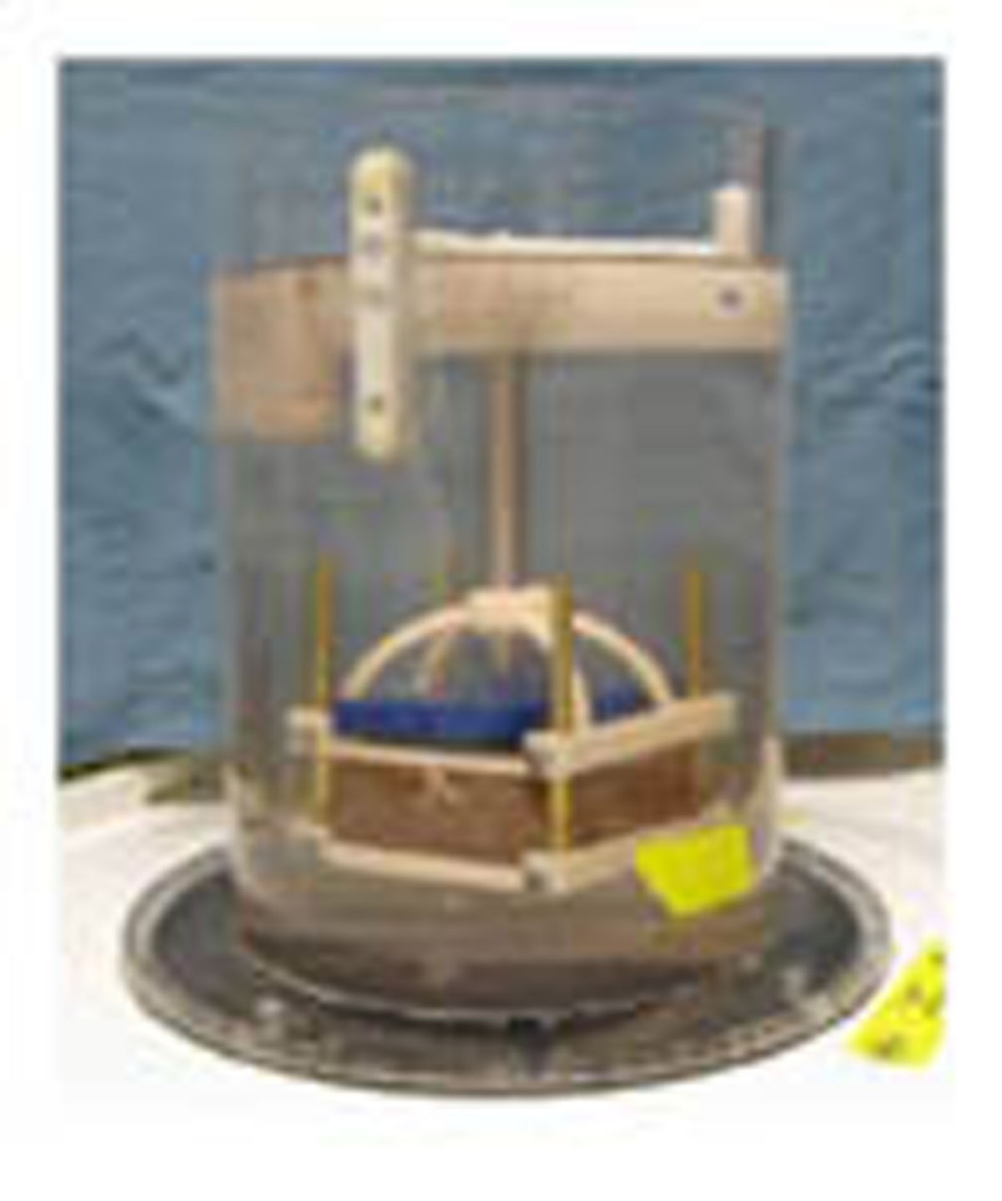
Photograph of the experimental setup at the patient table of the Sonalleve HIFU system for *ex vivo* tissue sonications.

**Figure 2:**
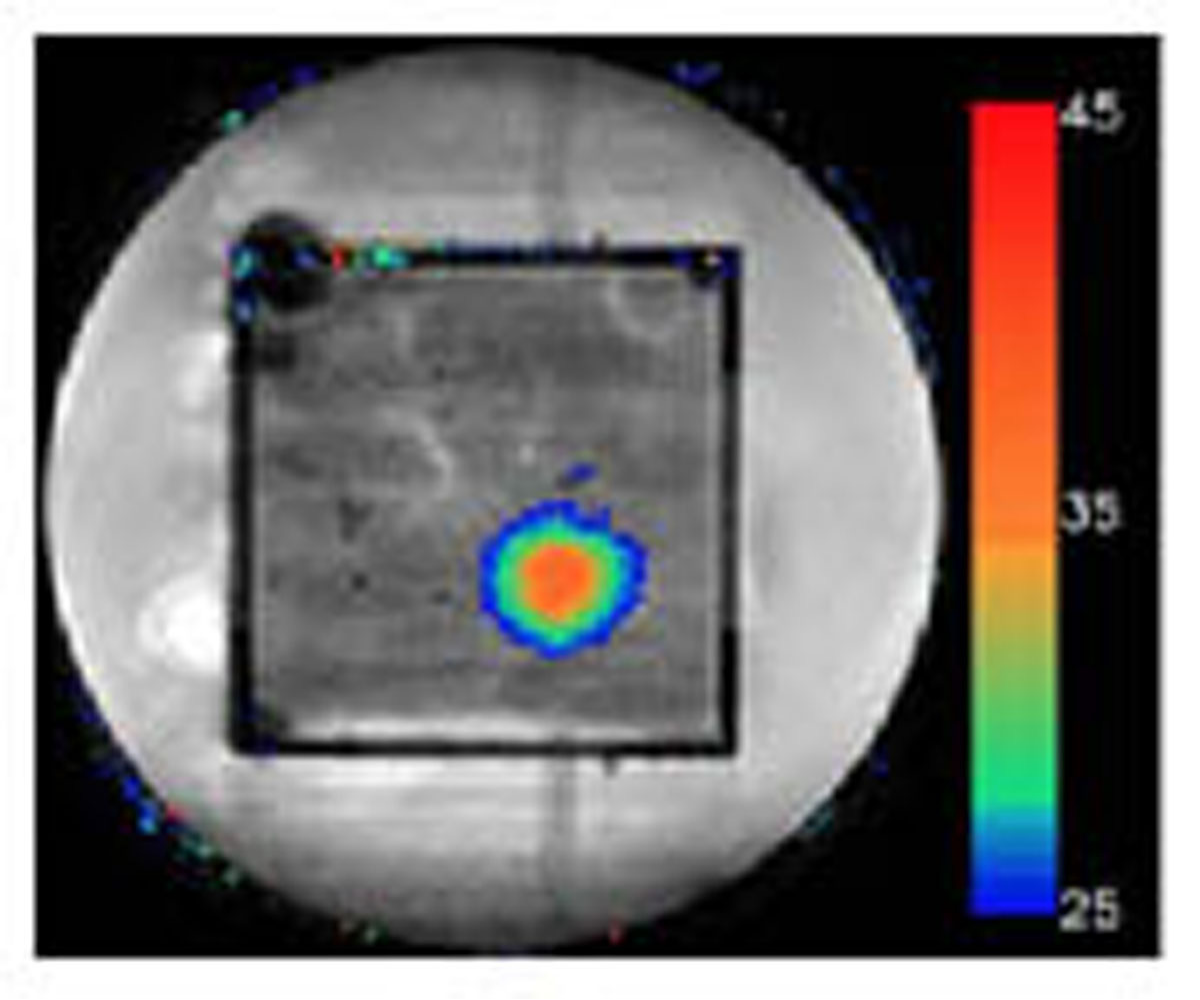
MR imaging of BH treatment in real time: temperature map in coronal imaging plane within the *ex vivo* bovine liver.

**Figure 3:**
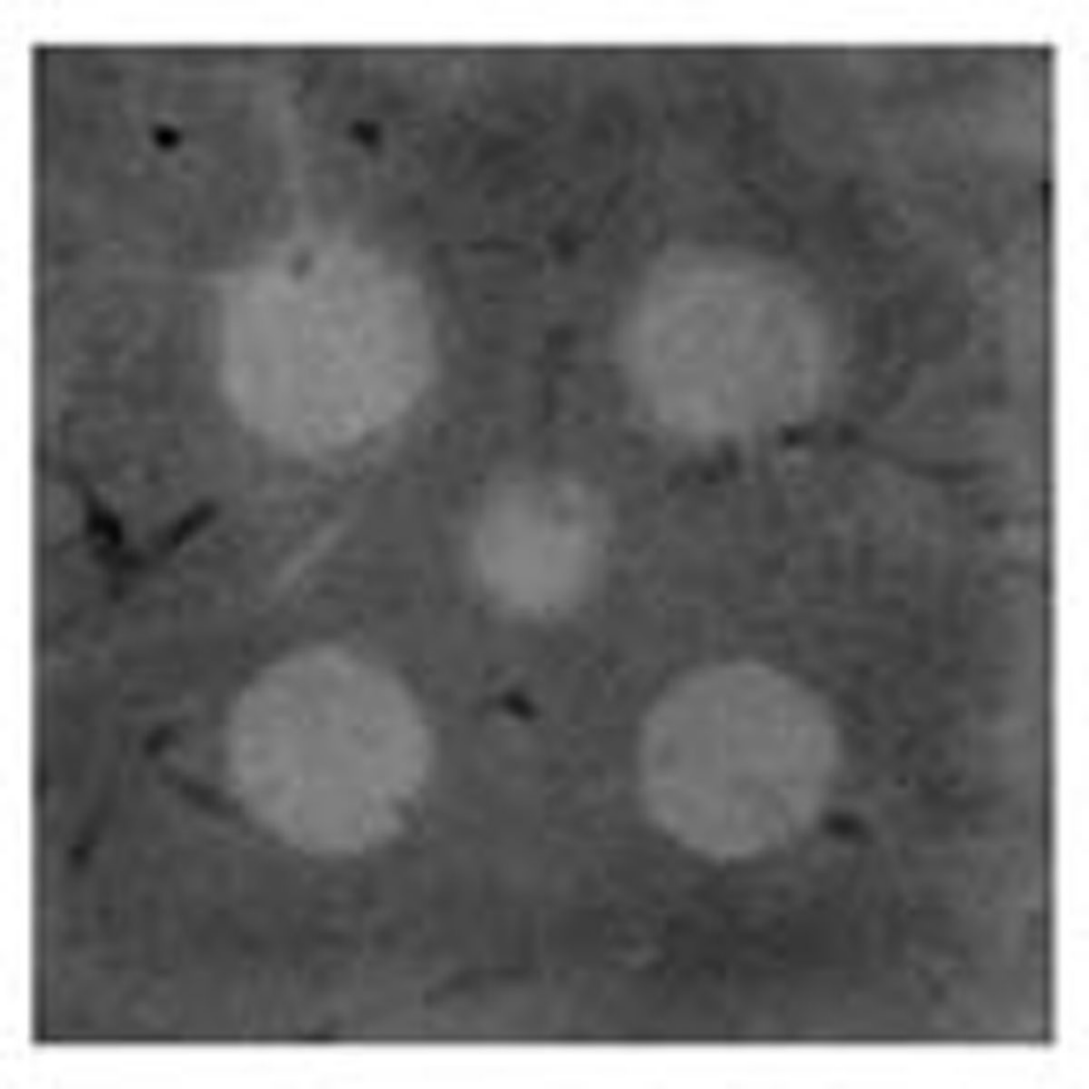
Post-treatment T2-weighted MR image of mechanically fractionated lesion in ex-vivo bovine liver.

**Figure 4:**
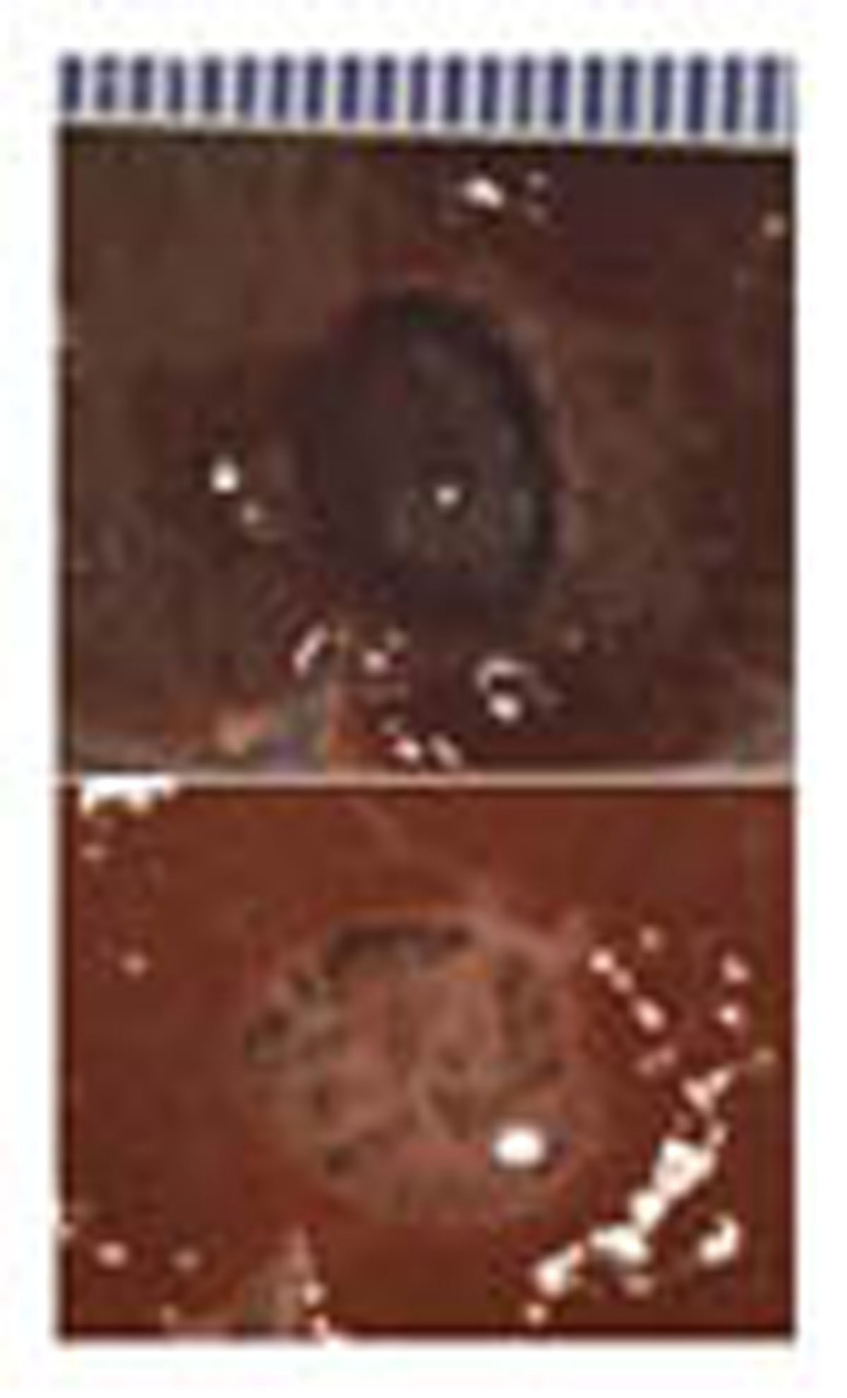
Mechanically fractionated volumetric lesions in *ex vivo* bovine liver with (top) and without (bottom) the content.

